# A New Formulation of Probiotics Attenuates Calcipotriol-Induced Dermatitis by Inducing Regulatory Dendritic Cells

**DOI:** 10.3389/fimmu.2021.775018

**Published:** 2021-11-16

**Authors:** Beilei Xu, Shiqi Ling, Xiaoqiang Xu, Xiaochun Liu, Ao Wang, Yuan Zhou, Yang Luo, Wei Li, Xu Yao

**Affiliations:** ^1^ Department of Allergy and Rheumatology, Jiangsu Key Laboratory of Molecular Biology for Skin Diseases and STIs, Hospital for Skin Diseases, Institute of Dermatology, Chinese Academy of Medical Sciences and Peking Union Medical College, Nanjing, China; ^2^ Department of Bioinformatics, 01life Institute, Shenzhen, China; ^3^ Department of Dermatology, Huashan Hospital, Fudan University, Shanghai, China

**Keywords:** probiotics, atopic dermatitis, regulatory T cells, regulatory dendritic cells, gut microbiota, short-chain fatty acids, butyrate

## Abstract

Atopic dermatitis (AD) is a recurrent chronic inflammatory skin disease affecting up to 30% of the children population, and immuno-regulatory therapy that could modify the course of disease is urgently needed. Probiotics have demonstrated therapeutic effects on AD and could potentially regulate the disease process. However, the efficacy of probiotics for AD is inconsistent among different studies, which is mainly due to the elusive mechanism and different species and (or) strains used. In this study, we designed a mixture of five strains of probiotics (named IW5) and analyzed the effect and mechanism of IW5 on calcipotriol (MC903)-induced AD-like dermatitis. We found that IW5 significantly alleviated skin inflammation of the MC903-induced AD in mice. Administration with IW5 induced increased production of regulatory T cells and regulatory dendritic cells (DCregs) in the mesenteric lymph nodes. We also found that the diversity of the gut microbiota in the mice with MC903-induced dermatitis was increased after IW5 administration, and the level of butyrate in the gut was elevated. In cell culture, butyrate induced the production of DCregs. Our study revealed the therapeutic effects of a newly designed probiotics mixture and uncovered a possible mechanism, providing a foundation for future clinical studies.

## Introduction

Atopic dermatitis (AD) is a common chronic inflammatory skin disease characterized by intensive itch, dry skin, and eczematous dermatitis (1). Barrier dysfunction, type 2-dominant skin inflammation, and skin/gut dysbiosis play important roles in the pathogenesis of AD. There has been great progress in the treatment of AD in recent years, for example, the biologics and Janus kinase inhibitors have demonstrated excellent efficacy in moderate to severe AD; however, AD is still refractory and often relapses when the treatment of biologics or JAK inhibitors is stopped. Thus, immuno-regulatory therapy that could modify the course of disease is urgently needed. Probiotics have potent immuno-regulatory effects and have shown preventive and therapeutic effects in inflammatory disorders by inducing the differentiation of regulatory T cells (Tregs) and balancing Th1/Th2 immune responses (2, 3). Many clinical studies have explored the efficacy of probiotics for the treatment of AD, and majority of the studies have demonstrated significant therapeutic effects on AD (4–11). However, meta-analyses on the clinical trials exploring the efficacy of probiotics in AD reveal that there is no significant difference between the group of probiotics and the control when the data are combined (12, 13). Whereas stratified analyses do show therapeutic effects of the probiotics, for example, Asian population, 1-18 years-old children and adolescents, and moderate to severe AD patients have significantly improved efficacy (14). The inconsistent results for probiotics might be because that the mechanism of the probiotics is not clear, and each probiotics species and (or) strain has different effect.

Several studies have explored the mechanism of probiotics for the treatment of allergic diseases. Administration with *Bifidobacterium* strain to mice results in increased expression of IL-10, TGF-β, IDO, and PD-1 within the mucosal CD103^+^ DCs, which, in turn, induce the development of Tregs (15). *Lactobacillus paracasei* L9 prevents food allergy in mice by inducing the production of regulatory dendritic cells (DCregs) and Foxp3^+^ Tregs (16). It has been reported that gut microbiota regulates immune responses through the production of short-chain fatty acids (SCFAs) such as acetate, propionate, and butyrate by fermentation of dietary fiber (17, 18). Microbiota-derived SCFAs promote CD103^+^ DCs to induce the differentiation of Tregs and increase the production of IL-10, suppressing colonic inflammation and carcinogenesis (19). It has been reported that the levels of SCFAs are decreased in the gut of AD patients (20). However, whether probiotics exert their function through the production of SCFAs is not clear.

In this study, we designed a mixture of probiotics by combining 5 strains of bacteria (named IW5) according to previous reports (21–26) and investigated the immuno-modulatory properties of IW5 in the calcipotril (MC903)-induced AD mouse model. We found that IW5 had significant therapeutic effects on the mouse model of AD, and administration with IW5 induced increased production of Tregs and DCregs in the mesenteric lymph nodes (MLNs). Our study revealed the effect and uncovered a possible mechanism of a newly designed formulation of probiotics, providing a foundation for future clinical studies.

## Materials and Methods

### Animals

Six to eight weeks-old female BALB/c mice were purchased from the Laboratory Animal Center of the Nanjing Medical University (Nanjing, Jiangsu, China). All the mice were maintained with a 12-h light/dark cycle at 22–24°C under specific pathogen-free condition. A standard extruded pellet diet and water were supplied unlimited. Animal experimental procedures were ethically reviewed and approved by the Animal Welfare Ethics Review Committee of the Institute of Dermatology, Chinese Academy of Medical Sciences.

### Probiotics Preparation

The probiotics mixture, Indiv Wellness (IW5**)**, was composed of five strains of probiotics: *Bifidobacterium lactis* UABLa-12, *Lactobacillus acidophilus* La-14, *Lactobacillus helveticus* R0052, *Lactobacillus salivarius* LS97, and *Lactobacillus casei* LC89. Probiotics were provided by 01life Institute (Shenzhen, Guangdong, China) as a lyophilized powder form, containing 5 × 10^10^ CFU active probiotics per gram.

### Mice Experiment

**Figure 1:**
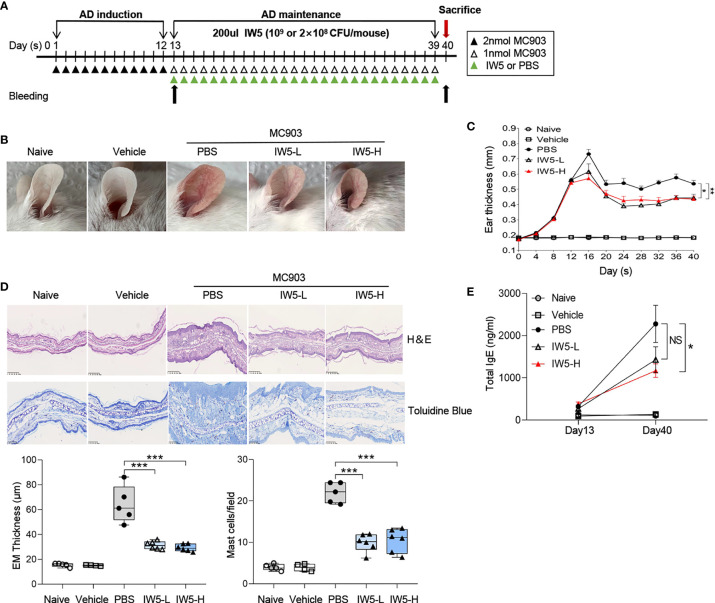
IW5 ameliorates MC903-induced AD symptoms in mice. **(A)** Experimental design. Mice were topically treated with MC903 for 12 days and then gavaged with IW5-L (2 × 10^8^ CFU IW5), or IW5-H (10^9^ CFU IW5) daily for 27 days. Oral feeding of PBS to the MC903-treated mice was used as control for probiotics, and mice with EtOH painted on the ears were used as vehicle control for the MC903-induced AD model. **(B)** Gross appearance of the ears on day 40. **(C)** Dynamic changes of ear thickness at the indicated time points. **(D)** Histology of skin lesion. Hematoxylin and eosin (H&E) staining shows the thickness of the epidermis (EM) (original magnification ×100, Scale bar = 250 μm), and toluidine blue staining shows the infiltration of mast cells (original magnification ×200, Scale bar = 100 μm). **(E)** Levels of serum total IgE at day 13 and day 40 as measured by ELISA. Data are shown as means ± SEMs, and representative of data from three independent experiments. Student’s t-test (unpaired; n = 5~6); *P < 0.05, **P < 0.01, ***P < 0.001 *vs.* PBS group. NS, not significant.

AD-like dermatitis was produced by topical application with 2 nmol of MC903 (calcipotriol; Leo Pharma, Ballerup, Denmark) in ethanol on each ear of mice once daily for 12 consecutive days as previously described (27). Once the dermatitis was fully induced, 1 nmol of MC903 was applied daily for 27 days to maintain the skin inflammation. At the same time, mice were intragastrically administered with 200 μl IW5-L (2 × 10^8^ CFU IW5) or IW5-H (1 × 10^9^ CFU IW5) daily for 27 days (**Figure 1A**), and intragastrical phosphate-buffered saline (PBS; Gibco, Carlsbad, CA, USA) was used as control. For prevention experiment, mice were first intragastrically treated with IW5-H or PBS daily for 23 days, then were topically treated with PBS or MC903 for 12 days (**Figure S1A**). At the time points indicated, full thickness of the ears was measured with a micrometer. At the end of the treatment, mice were euthanized using CO_2_. Skin tissue was either fixed in formalin for histopathological analysis or stored at -80°C for mRNA expression detection. Blood samples were collected for ELISA. The cervical lymph nodes (CLNs), MLNs, and spleens were harvested for further Fluorescence-activated cell sorting (FACS) analysis and cell culture.

### Histopathological Analysis

The ear of mice was cut into pieces, fixed in 4% formalin, and six micrometer sections were prepared and stained with hematoxylin and eosin (H&E) for evaluation of the epidermal thickness and inflammation. Sections were also stained with toluidine blue for analysis of mast cell infiltration.

### *Ex Vivo* Cell Culture and Treatment

CLNs, MLNs, and spleens were harvested and grinded with syringe pistons, and then were filtered through a 200-mesh strainer to get single-cell suspension for cell culture and flow cytometry. For isolation of splenocytes, red blood cells were removed by RBC lysis buffer (Miltenyi Biotec, Bergisch Gladbach, Germany). Single-cell suspensions of CLNs were resuspended in RPMI 1640 culture medium (Gibco) supplemented with 10% heat-inactivated fetal calf serum (FBS; Gibco), 100 U/ml penicillin, and 100 µg/ml streptomycin at a concentration of 10^6^ cells per milliliter and placed in a plate precoated with 2 μg/ml anti-CD3 and anti-CD28 monoclonal antibodies (mAbs; eBioscience, San Diego, CA, USA). Following 5 days of culture, the supernatants were harvested and stored at −80°C for IL-4, IFN-γ, IL-10, and TGF-β1 measurement using ELISA.

For experiments of CD4^+^ T Cells and CD11c^+^ DCs co-culture, CD4^+^ T cells were sorted from splenocytes of wild-type BALB/c mice by CD4 MicroBeads UltraPure kit (Miltenyi Biotec), and CD11c^+^ cells were sorted from the MLNs of mice that were intragastrically given 200 µl IW5-H (10^9^ CFU) daily for 20 days by CD11c-PE antibody (eBioscience) and PE MicroBeads UltraPure kit (Miltenyi Biotec). CD11c^+^ DCs (10^5^ cells/ml) were co-cultured with CD4^+^ naïve T cells (10^6^ cells/ml) for 5 days, in the presence of TGF-β1 (0.5 ng/ml; Peprotech, Rocky Hill, NJ, USA) and IL-2 (10 ng/ml; Peprotech) in RPMI 1640 culture medium supplemented with 10% heat-inactivated FBS, 100 U/ml penicillin and 100 µg/ml streptomycin. Then cells were subjected to CD4^+^ CD25^+^ Foxp3^+^ Tregs analysis by flow cytometry.

For the experiment of CD11c^+^ DC culture, CD11c^+^ DC cells were isolated from splenocytes of wild-type BALB/c mice as previously described. CD11c^+^ DCs (10^6^ cells/ml) were co-cultured with live IW5 (10^7^ CFU/ml), or SCFAs (Butyric, Acetic or Propionic; 1mmol/L; Sigma-Aldrich, St. Louis, MO, USA) for 24 h, in the presence of LPS (0.5ug/ml; Sigma-Aldrich) in RPMI 1640 culture medium supplemented with 10% heat-inactivated FBS. After incubation, the supernatants were harvested and examined for IL-6, IL-12p70, and IL-10 using ELISA, and the cells were analyzed for proliferation and expression of CD80, MHCII, and PD-L1 by flow cytometry.

### ELISA

For analysis of serum total IgE, 96-well microplate was pre-coated with 100 μl/well of purified rat anti‐mouse IgE (1 μg/mL) overnight at 4°C, then blocked with 200 μl/well of 10% FBS at room temperature for 30 minutes, followed by incubation with diluted sera at room temperature for 2 hours. Biotin rat anti‐mouse IgE (2 μg/mL) was added afterward. Then, after washing the plate was incubated with 100 μl/well of streptavidin-HRP. After 30 mins at room temperature, tetramethylbenzidine (TMB, Beyotime, Shanghai, China) was added and the reaction was stopped by adding 50 μl/well of 2M H_2_SO_4_. The absorbance was measured at wavelength 450 nm. The levels of IFN-γ, IL-4, IL-10, and TGF-β1 in the supernatants were measured using the ELISA kits (all from Fcmacs, FMS-ELM027, FMS-ELM004, FMS-ELM009, FMS-ELM029, Nanjing, Jiangsu, China); the levels of IL-6 and IL-12p70 in the supernatants were measured using the ELISA kits (all from NOVUS Biologicals, Littleton, Colorado, USA) according to the manufacturer’s instructions.

### Quantitative RealTime PCR (RT-qPCR)

Total RNA was extracted from the ear skin using TRIzol reagent (Invitrogen, Carlsbad, CA, USA), according to the manufacturer’s instructions. Complementary DNA (cDNA) was synthesized and the following mRNA expression of each gene was measured using AceQ qPCR SYBR Green Master Mix (Vazyme Biotech Co., Ltd, Nanjing, Jiangsu, China). Cycling condition was 95°C for 15s and 60°C for 30s for 40 cycles. Results were normalized to β-actin and was determined using 2^-ΔΔCT^ calculation method. Primer sequences were shown in **Supplementary Table 1**.

### Flow Cytometry

Single-cell suspension (10^6^ cells/tube) was incubated with anti-CD16/32 antibody to block Fc receptors for 15min at 4°C. For surface staining, the cells were stained with antibodies or matched isotype control for 20 min at 4°C in the dark. The antibodies included anti-mouse CD3-PECY7, CD4-FITC, CD8-Percpcy5.5, CD25-APC, CD11c-PE, CD86-APC, CD80-PerCP-eFlour710, PD-L1-PECY7, MHCII-Alexa Flour 700, MHCII- APC, and CD103-FITC antibodies (all from Biolegend, San Diego, CA, USA). For intracellular staining, cells were cultured in the presence of 2μl/ml cell stimulation cocktail (eBioscience) for 6 h. The cells were fixed and permeabilized using fixation and permeabilization solutions, then stained with anti-mouse Foxp3-PE, IL4-PE, IFN-γ-APC, IL10-PE antibodies (all from eBioscience), or isotype control for 30 min at 4°C in the dark. The cells were detected using BD FACSVerse (BD Biosciences, Franklin Lakes, NJ, USA) or Aurora (Cytek, Fremont, CA, USA), and data were analyzed using the FlowJo 10.0.7 software (Tree Star, Ashland, OR, USA).

### Microbiome Analysis

Fresh feces of mice were collected sterilely and stored at −80°C. Fecal DNA was extracted using a previously described method (28) and sequenced the V3 to V4 region of bacterial 16S rRNA genes in 2 × 250 bp paired-end (PE) mode on the NovaSeq platform (Illumina, USA) according to the manufacturer’s instructions. The V3–V4 region of bacterial 16S rRNA gene was amplified with a pair of region-specific primers (Forward: CCTACGGGNGGCWGCAG; Reverse: GACTACHVGGGTATCTAATCC). Sequencing libraries were generated using the TruSeq^®^ DNA PCR-Free Sample Preparation Kit (Illumina) following the manufacturer’s recommendations and index codes were added. The library quality was assessed on the Qubit@ 2.0 Fluorometer (Thermo Scientific) and Agilent Bioanalyzer 2100 system. Raw reads were joined using FLASH (v1.2.11) with default parameters and trimmed with Trimmomatic (v0.39) using specified settings (“ILLUMINACLIP:/share/app/Trimmomatic-0.39/adapters/TruSeq3-PE.fa:2:30:10 LEADING:3 TRAILING:3 SLIDINGWINDOW:4:20 MINLEN:200”). Processed data were then analyzed using QIIME-2020.2 software (https://docs.qiime2.org/2020.2/), and ASVs were analyzed using deblur method. Alpha diversity was evaluated using the Observed ASVs counts, Shannon index and Faith Phylogenetic Diversity (Faith PD), and Wilcoxon rank-sum test was conducted to identify statistical significance between two groups. Bray-Curtis distance measured at the ASV level was used to determine the dissimilarity of the microbial community, and intergroup comparison was conducted using PERMANOVA with 9999 permutations in the R package vegan. Generated ASVs were then further classified into taxonomy with q2-classify using the SILVA database (release 132). Kruskal-Wallis test was used to identify differential genus or ASVs between groups. A minimum occurrence frequency cutoff of 30% for each feature in either group was applied to filter genus or ASVs before differential analysis. P-values of less than 0.05 were considered to indicate significant results.

### SCFAs Quantitative Analysis

GC-MS analysis of SCFAs in the feces and serum of mice was performed on an Agilent HP-INNOWAX (Agilent Technologies Inc., Palo Alto, CA, USA) with capillary columns. Feces and serum was homogenized with 15% phosphoric acid, internal standard solution (Isocaproic acid), and diethyl ether, then centrifuged at 12,000 × g for 10 min at 4°C. The supernatants were then collected for GC-MS analysis.

### Statistical Analysis

The difference between individual pairs was calculated using Student’s t-test in the case of parameters. For nonparametric data, the Mann–Whitney test was used to determine statistical significance. All data were analyzed with GraphPad Prism V 8.0.1 and R 3.5.1 (GraphPad Software, La Jolla, CA, USA). Differences were considered statistically significant when P-value was <0.05.

## Results

### IW5 Alleviated Skin Inflammation in Mouse Model of AD

To investigate the effects of IW5 on AD, a mouse model of AD was produced by topical application with MC903; then two doses of IW5 were intragastrically administrated to the AD mice after 12 days of MC903 application (**Figure 1A**). The AD model induced by MC903 exhibited obvious skin inflammation manifesting as erythema, edema, crust, and scale, accompanied by increased levels of total serum IgE (**Figures 1B–E**). However, the AD mice receiving two doses of IW5 (IW5-L or IW5-H) showed significantly improved dermatitis, as evidenced by milder morphological appearance, decreased ear thickness, and less inflammatory cell infiltration (**Figures 1B–D**). The IW5-H group, but not the IW5-L group, also showed a decreased level of serum total IgE that was markedly elevated in the AD model mice (**Figure 1E**). Preventive effects of IW5 were further investigated, and the result showed that the mice receiving intragastrical IW5 first and topical MC903 later also demonstrated significantly decreased skin inflammation (**Figure S1**). Collectively, these data suggested that oral administration with the probiotics mixture IW5 alleviated MC903-induced AD-like dermatitis, in a dose-dependent manner.

### IW5 Inhibited Th2 Immune Responses in AD Model and Induced Tregs Production

**Figure 2:**
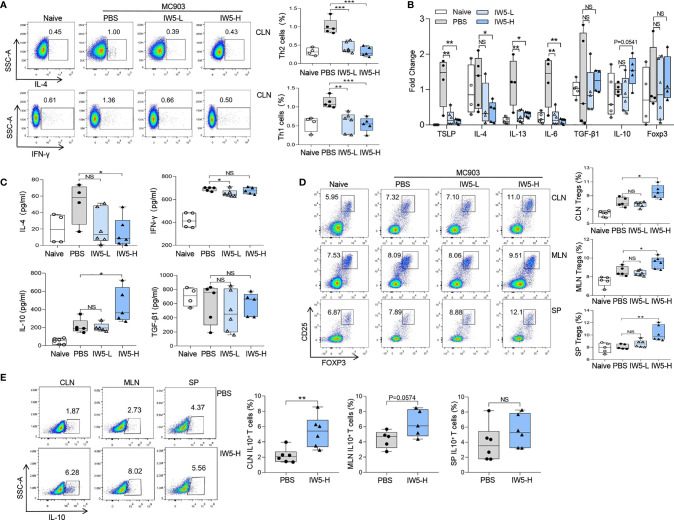
IW5 inhibits Th1 and Th2 polarization and promotes Tregs differentiation. **(A–D)** AD mice were orally administrated with IW5-L or IW5-H for 27 days, and the cervical lymph nodes (CLNs), mesenteric lymph nodes (MLNs), and spleens were collected on day 40 for further analysis. Th1 (CD4^+^ IFNγ^+^) and Th2 (CD4^+^ IL-4^+^) cells from the CLNs **(A)**, and Tregs (CD4^+^ CD25^+^ Foxp3^+^) from the CLNs, MLNs, and spleens **(D)** were analyzed by flow cytometry. RT-qPCR shows mRNA expression of various cytokines from the ear lesional skin **(B)**, and ELISA shows concentrations of IL-4, IFN-γ, IL-10, and TGF-β1 in supernatants of cells from the CLNs **(C)**. **(E)** BALB/c mice were administrated with IW5-H (10^9^ CFU IW5) or PBS daily for 20 days. Percentages of CD4^+^ IL-10^+^ T cells from the CLNs, MLNs, and spleens were measured by flow cytometry. Data are shown as means ± SEMs, and representative of data from three independent experiments. Student’s t-test (unpaired; n = 4~6); *P < 0.05, **P < 0.01, ***P < 0.001 *vs.* PBS group. NS, not significant.

Next, the differentiation and polarization of T cells in the AD mice after IW5 treatment were assessed. The proportions of Th1 (CD4^+^ IFN-γ^+^) and Th2 (CD4^+^ IL-4^+^) cells were increased in the CLNs of the AD mouse model; whereas in the AD mice receiving oral IW5, the percentage of Th1 cells (IW5-H *vs.* PBS; mean diff=-0.578%, P<0.001) and Th2 cells (IW5-H *vs.* PBS; mean diff=-0.68%, P<0.01) in the CLNs was significantly decreased (**Figures 2A**, **S2A, D**). The percentages of Th1 and Th2 cells in the MLNs (IW5-H *vs.* PBS; Th1 cells; mean diff=-0.094%, P>0.05; Th2 cells; mean diff=-0.254%, P>0.05) and spleens (IW5-H *vs.* PBS; Th1 cells; mean diff=-0.01% P>0.05; Th2 cells; mean diff=--0.156%, P>0.05) of the AD mice treated with IW5 were also lower than those of the AD model control, but the difference was not statistically significant (**Figures S2B, C**). The expression of TSLP, IL-4, IL-13, and IL-6 in skin lesions of the AD mice after IW5 treatment were markedly decreased (**Figures 2B**, **S2E**). Single cells from the CLNs of IW5-treated AD mice were then cocultured with anti-CD3 and anti-CD28 mAbs, and the levels of IL-4 and IFN-γ in the supernatant of culture were much lower than those of cells from the AD model control (**Figure 2C**). These data indicated that IW5 attenuated the polarization of Th1 and Th2 in AD mice.

The proportion of Tregs (CD4^+^ CD25^+^ Foxp3^+^) in the AD mice upon IW5 treatment was also analyzed. The result showed that the proportion of Tregs was significantly higher in the CLNs (mean diff=1.496%, P=0.0208), MLNs (mean diff=0.939%, P=0.0240), and spleens (mean diff=2.191%, P<0.01) of the AD mice receiving IW5-H compared to the AD mice receiving PBS (**Figures 2D** and **S2F**). The proportion of Tregs was also increased in the AD mice receiving IW5-L, but without statistical significance, indicating a dose-dependent effect of IW5. The concentration of IL-10 in the supernatants of CLN cell culture was increased in the IW5-H-treated AD mice compared with the AD model control, and the expression of IL-10 in the skin lesions of the IW5-H-treated AD mice was also elevated. However, there was no significant difference in the level of TGF-β1 between the IW5-treated AD mice and the AD model control (**Figures 2B, C**). These data indicated that the increased production of Tregs was induced through IL-10, but not TGF-β1.

We further analyzed CD4^+^ IL-10^+^ T cells in the naïve mice receiving oral IW5 and found that the percentages of CD4^+^ IL-10^+^ T cells in MLNs (mean diff=2.09%, P=0.0574) and CLNs (mean diff=3.153%, P<0.01) were higher in the IW5-treated mice compared to the PBS-treated mice (**Figures 2E**, **S2A**). The percentages of CD4^+^ IL-10^+^ T cells in the spleens (mean diff=1.615%, P>0.05) of the IW5-treated mice were also higher than those from the PBS-treated control mice, but the difference was not statistically significant (**Figure 2E**). Collectively, these results demonstrated that IW5 induced the differentiation of Tregs, and inhibited Th1 and Th2 immune responses in the mouse model of AD.

### IW5 Induced the Production of DCregs

**Figure 3:**
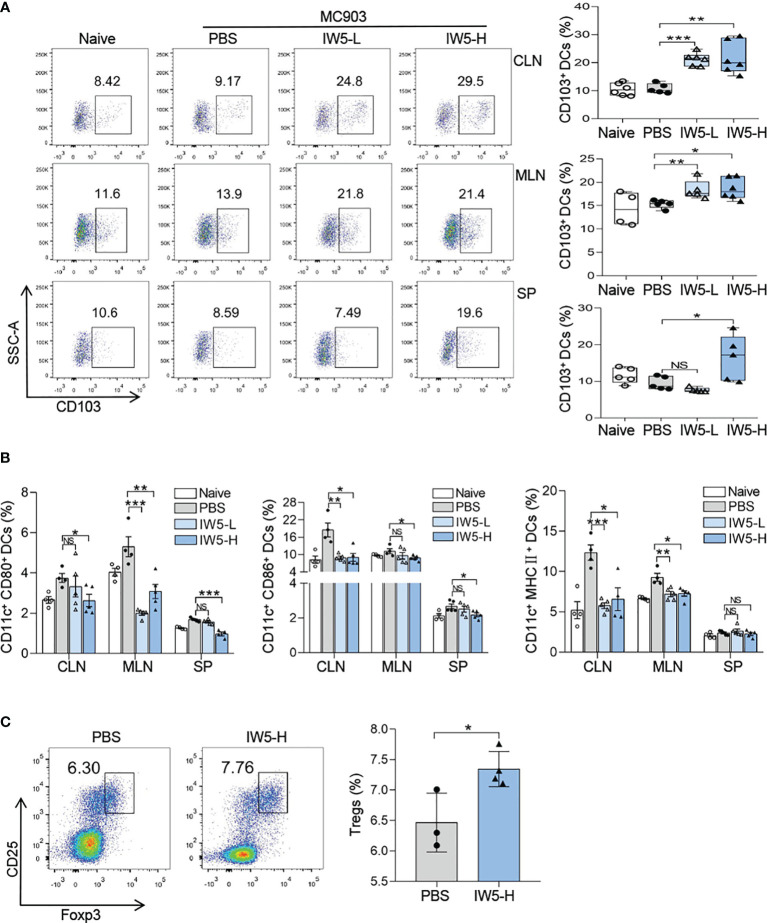
IW5 promotes DCregs expansion and Tregs differentiation. **(A, B)** AD mice were orally administrated with IW5-L or IW5-H daily for 27 days, and the CLNs, MLNs, and spleens were collected on day 40 for further analysis. CD103^+^ DCs (CD11c^+^ MHCII^+^ CD103^+^) were examined by flow cytometry **(A)**. Expression levels of CD80, CD86, and MHCII on CD11c^+^ DCs were examined by flow cytometry **(B)**. **(C)** BALB/c mice were orally administrated with IW5-H daily for 20 days. CD11c^+^ DCs from the MLNs were co-cultured with CD4^+^ T cells from naive mice spleens for 5 days. The proportion of CD4^+^ CD25^+^ Foxp3^+^ Tregs was measured by flow cytometry. Data are shown as means ± SEMs, and representative of data from three independent experiments. Student’s t-test (unpaired; n = 3~6); *P < 0.05, **P < 0.01, ***P < 0.001 *vs.* PBS group. NS, not significant.

CD103^+^ DCs are considered as the iconic mucosal DCregs, which induce the production of Foxp3^+^ Tregs (29). We then analyzed the number of DCregs in naïve mice after IW5 administration. The result of FACS showed that the percentage of CD103^+^ DCs (CD11c^+^ MHCII^+^ CD103^+^) was increased in the MLNs (IW5-H *vs.* PBS; mean diff=3.26%, P=0.0164; IW5-L *vs.* PBS; mean diff=3.04%, P=0.0149), CLNs (IW5-H *vs.* PBS; mean diff=11.019%, P<0.01; IW5-L *vs.* PBS; mean diff=10.385%, P<0.001) of the mice treated with IW5-H or IW5-L (**Figures 3A**, **S3A**). Whereas in the spleens, the percentage of CD103^+^ DCs (IW5-H *vs.* PBS; mean diff=6.894%, P=0.0449) was increased only in the IW5-H-treated mice (**Figure 3A**). The expression of CD80, CD86, and MHCII on CD11c^+^ DCs from the mice treated with IW5-H was lower than those of the cells from the mice treated with PBS (**Figure 3B**). CD11c^+^ DCs were next isolated from the spleens of naïve mice and cultured in the presence of IW5 for 24 h, and the number of CD103^+^ DCs and the concentration of IL-10 in the supernatant were increased significantly (**Figures S3B, C**). CD11c^+^ DCs isolated from the MLNs of IW5-H treated mice were cocultured with naïve CD4^+^ T cells isolated from naïve mice spleens for 5 days, and the number of Tregs (IW5-H *vs.* PBS; mean diff=0.878%, P=0.0289) was higher compared with those cocultured with CD11c^+^ DCs from the PBS-treated mice (**Figure 3C**). Collectively, these results demonstrated that IW5 induced the expansion of DCregs *in vivo* and *in vitro*, which promoted the differentiation of Tregs.

### IW5 Increased Gut Microbiota Diversity and SCFAs Production in AD Mouse Model

**Figure 4:**
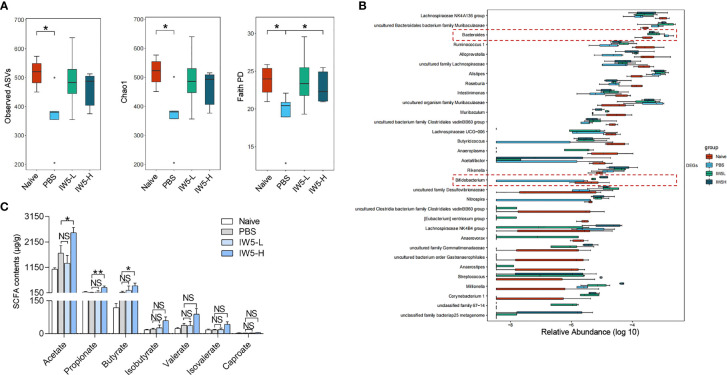
IW5 modulates fecal microbiota composition and SCFAs production. Mice feces were collected from the MC903-induced AD model before and after IW5 treatment. **(A)** Alpha-diversity of the gut microbiota as indicated by the observed ASVs, chao1 index, and faith pd index. **(B)** Relative abundance of the gut microbiota at the genus level. **(C)** Contents of acetate, propionate, butyrate, isobutyrate, valerate, isovalerate, and caproate in fecal samples. Data are shown as means ± SEMs, and representative of data from three independent experiments. Student’s t-test for parameters, and Mann-Whitney test for nonparametric data. (unpaired; n = 4~6); *P < 0.05, **P < 0.01 *vs.* PBS group. NS, not significant.

Next, we explored the effects of IW5 on the gut microbiota and their metabolites, SCFAs, in the mouse model with AD (30–33). Mice feces were collected before and after IW5 treatment in the MC903-induced AD model, and 16s rRNA was amplified and sequenced for analysis of the community structure and specific microbes of the gut microbiota. The result showed that the alpha diversity of the AD model mice was increased after oral administration with IW5, as indicated by the observed ASVs, chao1 index, and faith pd index (**Figure 4A**). Principal coordination (PCoA) analysis based on the ASV levels further demonstrated the changes in the microbial composition (**Figure S4A**). At the genus level, the abundance of *Bacteroides* was decreased and that of *Bifidobacterium* was increased in the AD model mice after oral IW5 administration (**Figure 4B**). Furthermore, the levels of acetate, propionate, and butyrate in the feces of the IW5-H-treated AD mice were significantly higher than those from the feces of the control AD mice. The levels of isobutyrate, valerate, isovalerate, and caproate had the same trend of increase but did not show statistical differences (**Figures 4C**, **S4B**), and there was no difference in serum level of SCFAs between IW5-treated mice and MC903 control mice (**Figure S4C**). These data demonstrated that IW5 restored the decreased diversity of the gut microbiota in the AD mice induced by MC903 application and increased the production of SCFAs.

### Butyrate Induced the Production of DCregs *In Vitro*

**Figure 5:**
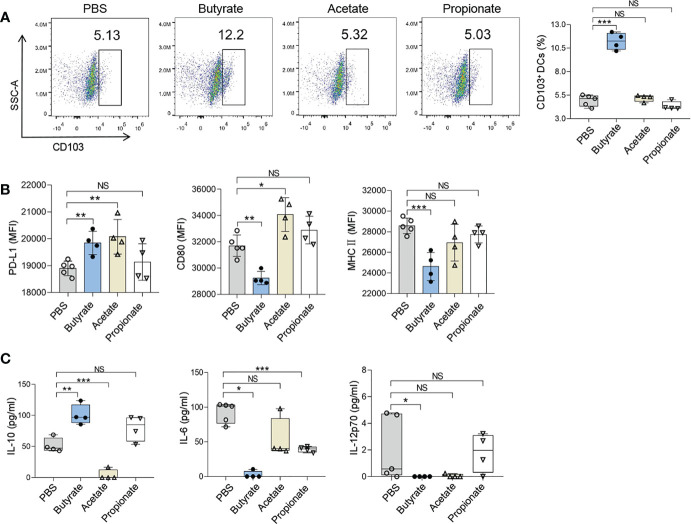
Butyrate promotes the *ex vivo* induction of DCregs. CD11c^+^ DCs from the spleen of naive mice were incubated with SCFAs for 24 h. **(A)** Numbers of CD103^+^ DCs (CD11c^+^ MHCII ^+^ CD103^+^) were measured by flow cytometry. **(B)** MFIs of CD80, MHCII, or PD-L1 on CD11c^+^ DCs were measured by flow cytometry. **(C)** Levels of IL-6, IL-10, and IL-12p70 in the supernatants of CD11c^+^ DCs were measured by ELISA. Data are shown as means ± SEMs, and representative of data from three independent experiments. Student’s t-test (unpaired; n = 4~5); *P < 0.05, **P < 0.01, ***P < 0.001 *vs.* PBS group. NS, not significant.

The effects of butyrate on DCregs and Tregs were also explored. CD11c^+^ DCs were isolated from the spleens of naive mice and were treated with 1mmol/L SCFA (acetate, propionate, or butyrate respectively) in culture for 24 h. The proportion of DCregs was significantly increased after butyrate treatment as shown by the result of flow cytometry analysis (**Figure 5A**). The proportion of CD103^+^ DCs (Butyrate *vs.* PBS; mean diff=6.297%, P<0.001) and expression level of PD-L1 on CD11c^+^ DCs in the presence of butyrate were higher than those of cells cultured in control medium, and the concentration of IL-10 in culture supernatant was also increased (**Figures 5B, C**). Whereas acetate and propionate didn’t show any effects on CD103^+^ DCs. These results indicated that butyrate was the key immuno-modulatory metabolite during IW5 treatment.

## Discussion

**Figure 6:**
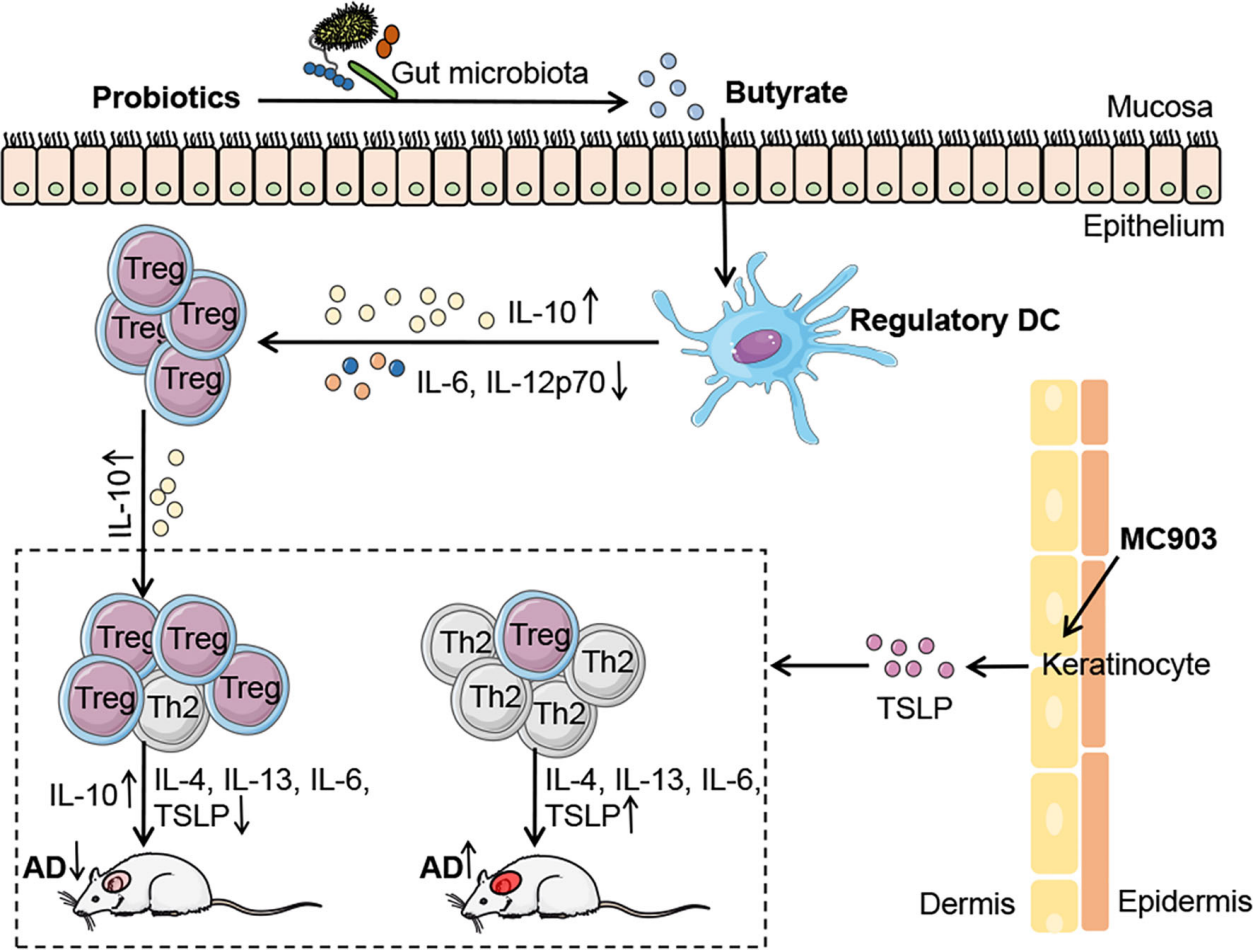
**|** Schematic illustrating the underlying mechanism of probiotics in AD. IW5 administration restores the diversity of the gut microbiota in AD mouse models and increases the production of SCFAs (acetate, propionate, and butyrate). Butyrate induces DCregs production, which results in Tregs differentiation and amelioration of AD symptoms.

Extensive studies have proven that probiotics have potent immuno-modulatory effects and have been used in the prevention and treatment of many diseases (34); however, the role of probiotics in allergic diseases, such as AD, remains poorly understood. In the present study, we demonstrated that the probiotics mixture IW5 had significant therapeutic and preventive effects on a mouse model of AD-like dermatitis, by inducing DCregs and Tregs. We also found that oral IW5 administration increased the diversity of the gut microbiota in the AD model and induced the production of butyrate that promoted the differentiation of DCregs (**Figure 6**). Our study revealed the therapeutic effects of a newly designed probiotics mixture that play a regulatory role in skin inflammation.

We first assessed the effects of IW5, an innovative combination of probiotics, on the MC903-induced AD mouse model. We compared the effects of two concentrations of IW5 and found that the high-dose probiotics exerted more pronounced effects on skin inflammation (**Figures 1A, E**), indicating that the effect of IW5 is dose-dependent. The most appropriate dose of probiotics still needs further exploration. A mixture of multiple strains of probiotics, instead of one probiotics strain, was used in our study, which might have advantages for the immuno-regulatory effects. Previous studies have demonstrated that multi-strain probiotics have optimal effects compared to single-strain probiotics (35). A mixture of selected strains of probiotics (IRT5) have shown more potent efficacy for disease control than single-strain probiotics (36). The enhanced benefits of multi-strain probiotics may be due to the complementary effects of constituent strains, additive and synergistic effects, and the cell-cell communication known as quorum sensing (37–40). The detailed mechanism of multiple strains of probiotics is still awaiting further exploration.

Probiotics have been reported to mitigate allergic diseases through the induction of Tregs, inhibition of Th2 immune responses, and promotion of intestinal DCregs expansion (2, 41, 42). Although innate immune responses play an important role in the MC903-induced AD-like dermatitis, in which TSLP and IL-33 produced by keratinocytes initiate the skin inflammation, adaptive immune cells are also required in the inflammation. Studies have demonstrated that the MC903-induced chronic inflammation is CD4^+^ T cell-dependent (43), and Tregs are involved in the skin inflammation and play a regulatory role (44). Thus, Tregs might mediate the immuno-regulatory effects of DCregs in our study. DCregs, a type of semi-mature DCs, are the pivotal regulator of immune tolerance (45–47) and exhibit a tolerogenic phenotype that includes downregulation of co-stimulatory molecules (CD80, CD86) and upregulation of the immuno-modulatory molecule PD-L1 (48, 49). In recent years, CD103^+^ DCs are considered the iconic mucosal DCregs, which have been shown to promote the differentiation of Tregs (50–52). We first found that IW5 administration promoted the production of DCregs *in vivo* and *in vitro* (**Figures 3A, B**, **S3B, C**), and *in vitro* co-culture experiment further showed that DCregs induced by IW5 promoted the induction of Tregs (**Figure 3C**). These results indicate that IW5 may alleviate AD symptoms *via* induction of DCregs and regulation of T cell immune responses, which resulted in an increased proportion of Tregs (**Figure 2B**) and decreased polarization of Th2 and Th1 cells (**Figure 2A**).

Previous studies have reported that the gut microbiota is closely related to allergic diseases (53). IW5 treatment modulated the structure of the gut microbiota, which might account for the therapeutic effects of IW5 on the MC903-induced AD model (**Figures 4A, B**, **S4A**). The abundance of the genus *Bifidobacterium* was significantly decreased in the MC903-induced AD mouse model and was positively correlated with the Treg/Th2 ratio; while the genus *Bacteroides* was markedly increased in the AD model and was negatively correlated with the Treg/Th2 ratio. These changes were reversed by IW5 administration (**Figure 4B**). Our result is consistent with previous data highlighting the regulatory role of *Bifidobacterium* and *Bacteroides* in AD and (or) food allergy patients. For example, less colonized *Bifidobacteria* during the first year of life is associated with infants’ allergic diseases (54); and the abundance of *Bifidobacterium* is an important determinant of Tregs maturation during early infancy (55). Conversely, some bacteria such as several strains of *Bacteroides* might increase the gut permeability and allergens exposure (56). The elevated relative abundance of *Bacteroides* species is also related to the non-IgE-mediated Cow’s milk allergy, nut allergy, peanut allergy, or other atopic symptoms (57–60). Notably, IW5 administration did not increase all the components of IW5, but modified other bacteria of the gut microbiota, indicating an indirect effect of IW5 in the modulation of the gut microbiota.

SCFAs have strong immuno-regulatory effects in allergic diseases (2, 50, 61), and butyrate is one of the key immuno-regulatory metabolites for the differentiation of Tregs (31). We found that IW5 increased the production of SCFAs, including acetate, propionate, and butyrate (**Figure 4C**). *In vitro* co-culture experiment showed that butyrate increased the percentages of DCregs, whereas acetate and propionate did not have this effect (**Figures 5A, B**). These results suggest that IW5 might exert its effect *via* the induction of butyrate. Our *in vitro* study also showed that culture of IW5 with DCs resulted in increased number of DCregs, indicating that bacteria antigen might also induce the differentiation of DCregs (**Figure S3**), which is consistent with previous report that heat-killed probiotics induce CD103^+^ DCs and attenuate inflammation (62, 63). As there is no difference in serum level of SCFAs between probiotics-treated mice and MC903 control mice (**Figure S4C**), it’s probable that SCFAs might exert their effects in the gut, inducing the production of DCregs and Tregs, and the latter would migrate to other organs and tissue. Future work should pay more attention to the metabolites of the gut microbiota. Supplementing specific microbiota that produces beneficial metabolites and (or) developing drugs from metabolites would be a promising strategy for the regulation of local and (or) systemic inflammation.

## Data Availability Statement

The original contributions presented in the study are included in the article/**Supplementary Material**. The raw reads for 16S rRNA Sequencing were deposited in ENA under accession number PRJEB47618.

## Ethics Statement

The animal study was reviewed and approved by the Animal Welfare Ethics Review Committee of the Institute of Dermatology, Chinese Academy of Medical Sciences.

## Author Contributions

BX and SL performed the experiments and analyzed the data. XX, XL, AW, YZ, and YL contributed to analysis the experimental work. BX, XY, and WL designed the experiments and wrote the manuscript. XY coordinated the research. All authors contributed to the article and approved the submitted version.

## Funding

This work was supported by the Natural Science Foundation of China (82073446, 81972939, 81803144, 81703126), the Nanjing Incubation Program for National Clinical Research Centre (2019060001), the Key Project of Social Development in Jiangsu Province (BE2020632), the Medicine and Health Technology Innovation Project of Chinese Academy of Medical Sciences (2016-I2M-1-005), the Key Project of the Innovation Program of Shanghai Municipal Education Commission (2021-01-07-00-07-E00078), and the Milstein Medical Asian American Partnership Foundation (WL).

## Conflict of Interest

Author XX is employed by 01life Institute. 

The remaining authors declare that the research was conducted in the absence of any commercial or financial relationships that could be construed as a potential conflict of interest.

## Publisher’s Note

All claims expressed in this article are solely those of the authors and do not necessarily represent those of their affiliated organizations, or those of the publisher, the editors and the reviewers. Any product that may be evaluated in this article, or claim that may be made by its manufacturer, is not guaranteed or endorsed by the publisher.
