# Supplementary material for: A New Formulation of Probiotics Attenuates Calcipotriol-Induced Dermatitis by Inducing Regulatory Dendritic Cells
Source: Front Immunol. 2021 Nov 16;12:775018. doi: 10.3389/fimmu.2021.775018 (PMC8634942; doi:10.3389/fimmu.2021.775018)
Supplement: Supplementary file 1 [file Presentation_1.pptx]

## Slide 1
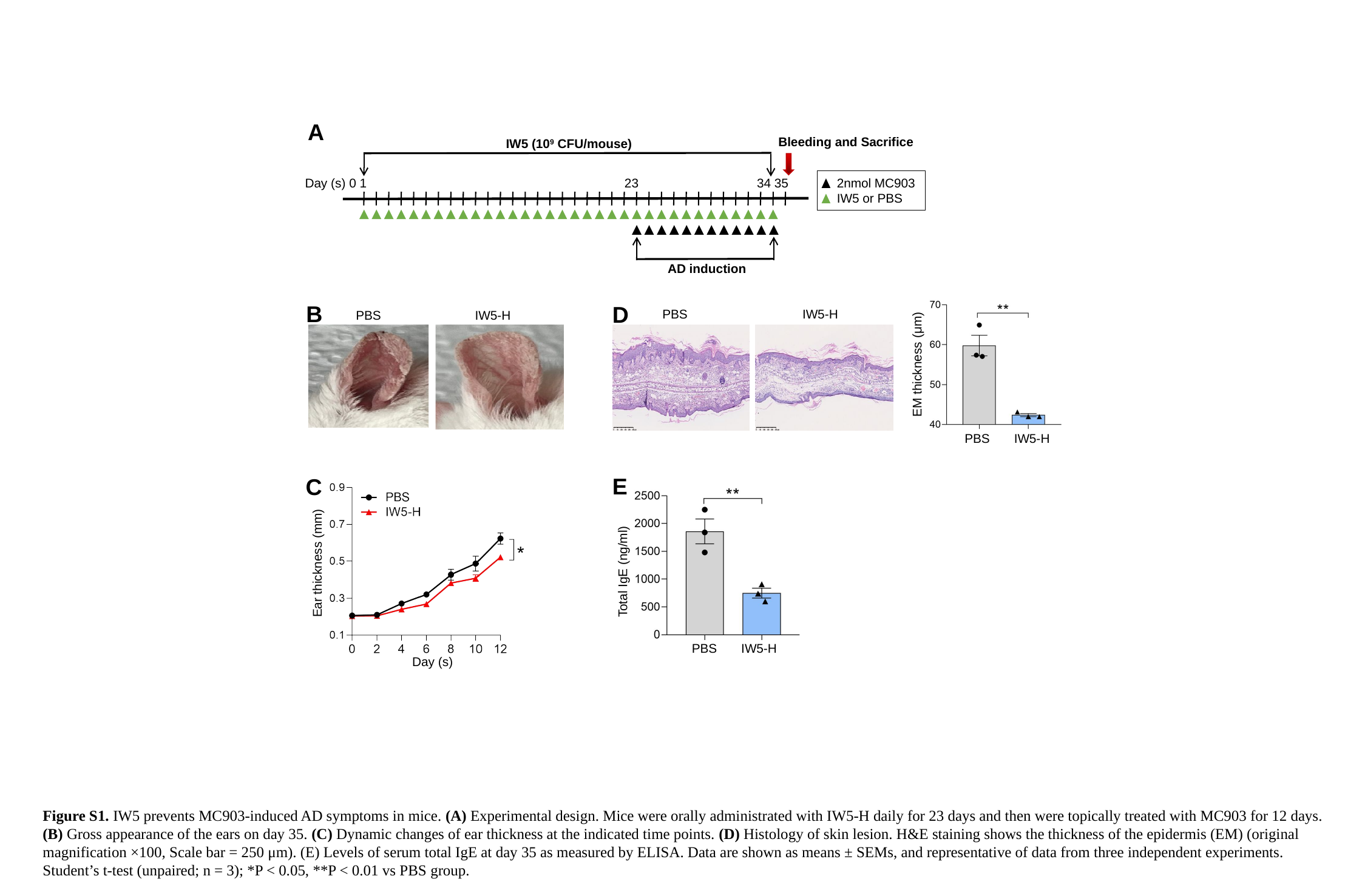

A
IW5 (109 CFU/mouse)
Bleeding and Sacrifice
 Day (s) 0 1 23 34 35
 2nmol MC903
 IW5 or PBS
AD induction
EM thickness (μm)
PBS IW5-H
D
 PBS IW5-H
B
 PBS IW5-H
E
Total IgE (ng/ml)
PBS IW5-H
C
Ear thickness (mm)
Day (s)
Figure S1. IW5 prevents MC903-induced AD symptoms in mice. (A) Experimental design. Mice were orally administrated with IW5-H daily for 23 days and then were topically treated with MC903 for 12 days. (B) Gross appearance of the ears on day 35. (C) Dynamic changes of ear thickness at the indicated time points. (D) Histology of skin lesion. H&E staining shows the thickness of the epidermis (EM) (original magnification ×100, Scale bar = 250 μm). (E) Levels of serum total IgE at day 35 as measured by ELISA. Data are shown as means ± SEMs, and representative of data from three independent experiments. Student’s t-test (unpaired; n = 3); *P < 0.05, **P < 0.01 vs PBS group.

## Slide 2
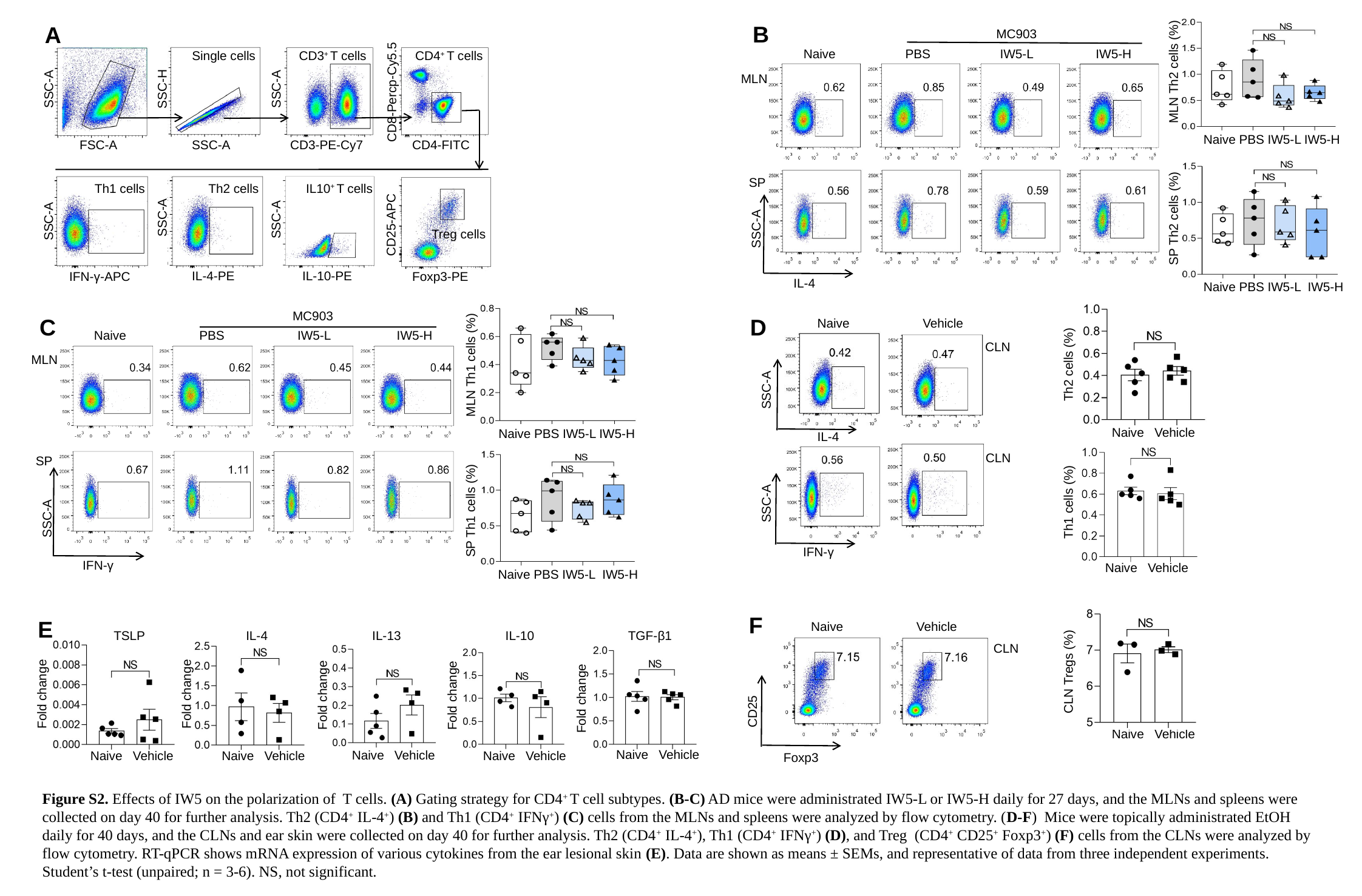

MLN Th2 cells (%)
Naive PBS IW5-L IW5-H
SP Th2 cells (%)
Naive PBS IW5-L IW5-H
B
MC903
Naive PBS IW5-L IW5-H
MLN
SP
SSC-A
IL-4
A
CD4+ T cells
CD8-Percp-Cy5.5
CD4-FITC
Single cells
SSC-H
SSC-A
CD3+ T cells
SSC-A
CD3-PE-Cy7
SSC-A
FSC-A
Th1 cells
SSC-A
IFN-γ-APC
SSC-A
IL-4-PE
Th2 cells
 IL10+ T cells
SSC-A
IL-10-PE
Treg cells
CD25-APC
Foxp3-PE
D
Naive Vehicle
CLN
Th2 cells (%)
SSC-A
IL-4
CLN
SSC-A
IFN-γ
Th1 cells (%)
 Naive Vehicle
 Naive Vehicle
MLN Th1 cells (%)
Naive PBS IW5-L IW5-H
SP Th1 cells (%)
Naive PBS IW5-L IW5-H
MC903
 Naive PBS IW5-L IW5-H
MLN
SP
SSC-A
IFN-γ
C
CLN Tregs (%)
 Naive Vehicle
F
 Naive Vehicle
CLN
CD25
Foxp3
E
 TSLP IL-4 IL-13 IL-10 TGF-β1
Fold change
 Naive Vehicle
Fold change
 Naive Vehicle
Fold change
 Naive Vehicle
Fold change
 Naive Vehicle
Fold change
 Naive Vehicle
Figure S2. Effects of IW5 on the polarization of T cells. (A) Gating strategy for CD4+ T cell subtypes. (B-C) AD mice were administrated IW5-L or IW5-H daily for 27 days, and the MLNs and spleens were collected on day 40 for further analysis. Th2 (CD4+ IL-4+) (B) and Th1 (CD4+ IFNγ+) (C) cells from the MLNs and spleens were analyzed by flow cytometry. (D-F) Mice were topically administrated EtOH daily for 40 days, and the CLNs and ear skin were collected on day 40 for further analysis. Th2 (CD4+ IL-4+), Th1 (CD4+ IFNγ+) (D), and Treg (CD4+ CD25+ Foxp3+) (F) cells from the CLNs were analyzed by flow cytometry. RT-qPCR shows mRNA expression of various cytokines from the ear lesional skin (E). Data are shown as means ± SEMs, and representative of data from three independent experiments. Student’s t-test (unpaired; n = 3-6). NS, not significant.

## Slide 3
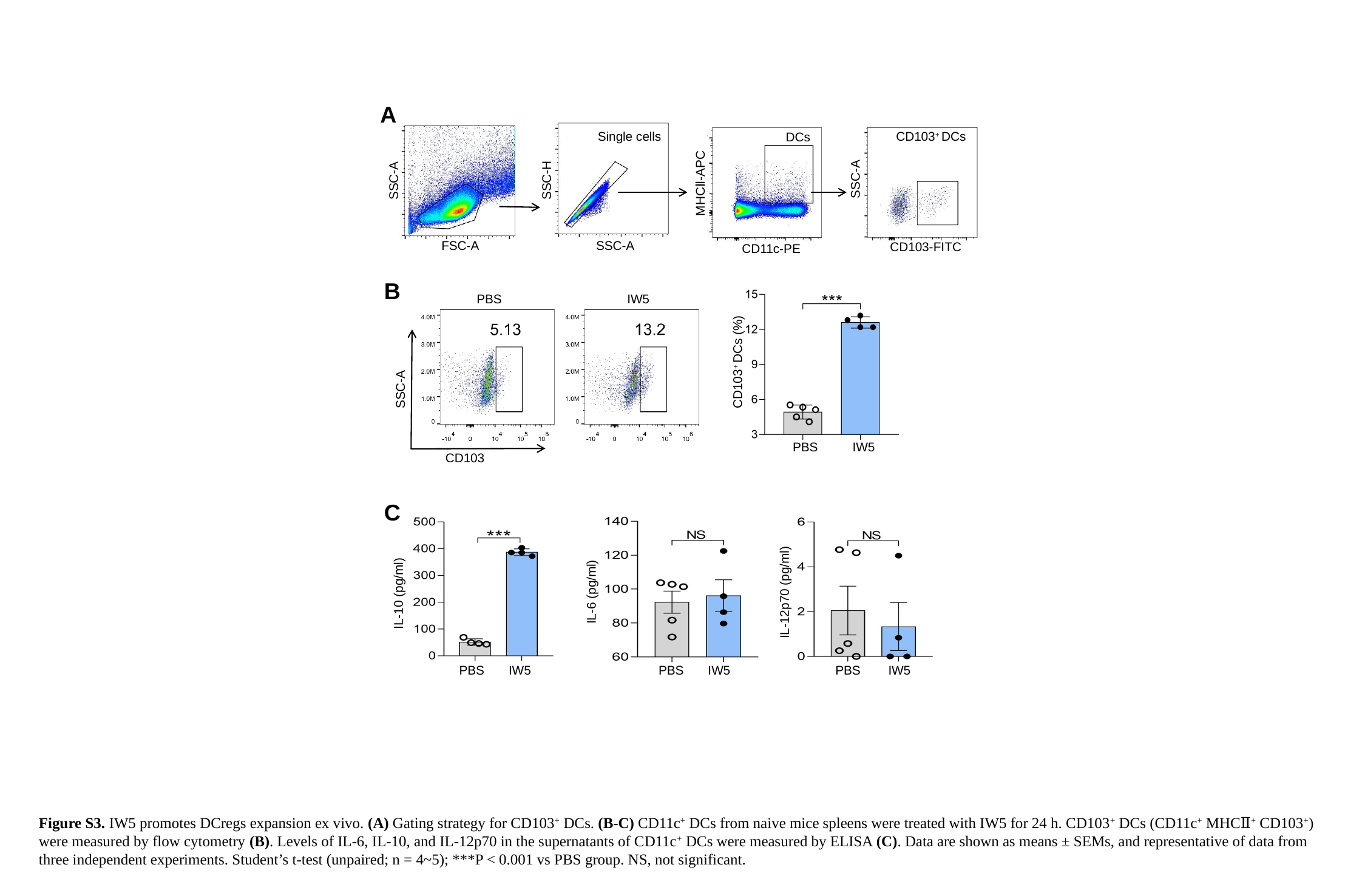

A
Single cells
SSC-H
SSC-A
SSC-A
FSC-A
CD103+ DCs
SSC-A
CD103-FITC
DCs
MHCⅡ-APC
CD11c-PE
B
 CD103+ DCs (%)
 PBS IW5
 PBS IW5
SSC-A
CD103
C
IL-10 (pg/ml)
 PBS IW5
IL-6 (pg/ml)
 PBS IW5
IL-12p70 (pg/ml)
 PBS IW5
Figure S3. IW5 promotes DCregs expansion ex vivo. (A) Gating strategy for CD103+ DCs. (B-C) CD11c+ DCs from naive mice spleens were treated with IW5 for 24 h. CD103+ DCs (CD11c+ MHCⅡ+ CD103+) were measured by flow cytometry (B). Levels of IL-6, IL-10, and IL-12p70 in the supernatants of CD11c+ DCs were measured by ELISA (C). Data are shown as means ± SEMs, and representative of data from three independent experiments. Student’s t-test (unpaired; n = 4~5); ***P < 0.001 vs PBS group. NS, not significant.

## Slide 4
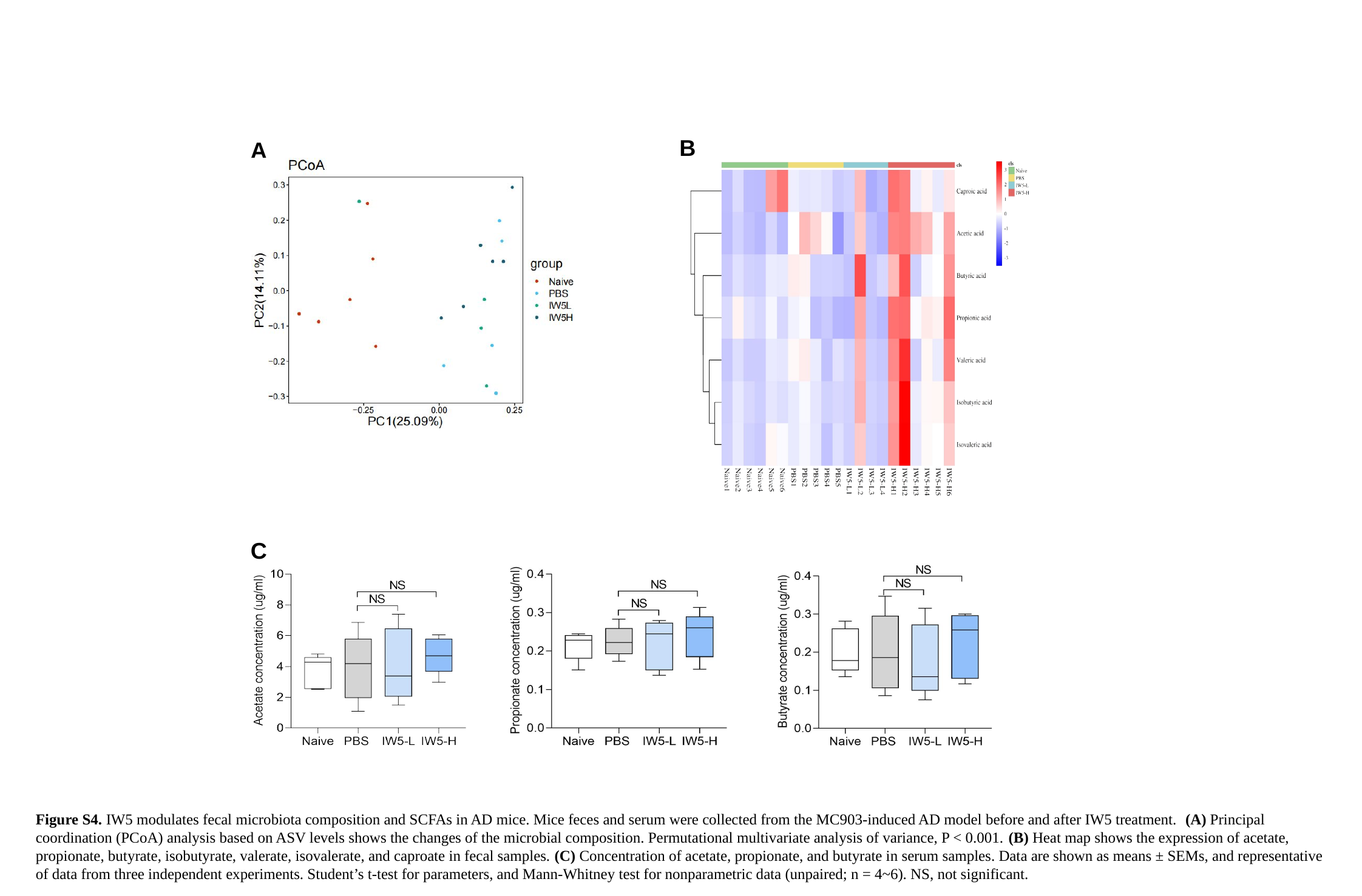

B
A
C
Figure S4. IW5 modulates fecal microbiota composition and SCFAs in AD mice. Mice feces and serum were collected from the MC903-induced AD model before and after IW5 treatment. (A) Principal coordination (PCoA) analysis based on ASV levels shows the changes of the microbial composition. Permutational multivariate analysis of variance, P < 0.001. (B) Heat map shows the expression of acetate, propionate, butyrate, isobutyrate, valerate, isovalerate, and caproate in fecal samples. (C) Concentration of acetate, propionate, and butyrate in serum samples. Data are shown as means ± SEMs, and representative of data from three independent experiments. Student’s t-test for parameters, and Mann-Whitney test for nonparametric data (unpaired; n = 4~6). NS, not significant.
