# Supplementary material for: A New Formulation of Probiotics Attenuates Calcipotriol-Induced Dermatitis by Inducing Regulatory Dendritic Cells
Source: Front Immunol. 2021 Nov 16;12:775018. doi: 10.3389/fimmu.2021.775018 (PMC8634942; doi:10.3389/fimmu.2021.775018)
Supplement: Supplementary file 2 [file Table_1.docx]

Primer sequences were shown in Supplementary Table 1:

| β-actin | F | GTGACGTTGACATCCGTAAAGA |
| --- | --- | --- |
|  | R | GCCGGACTCATCGTACTCC |
| TSLP | F | ACGGATGGGGCTAACTTACAA |
|  | R | AGTCCTCGATTTGCTCGAACT |
| IL-2 | F | GTGCTCCTTGTCAACAGCG |
|  | R | GGGGAGTTTCAGGTTCCTGTA |
| IFN-γ | F | ATGAACGCTACACACTGCATC |
|  | R | CCATCCTTTTGCCAGTTCCTC |
| TNF-a | F | CATCTTCTCAAAATTCGAGTGACA A |
|  | R | TGG GAG TAG ACA AGGTACAACCC |
| IL-4 | F | GGTCTCAACCCCCAGCTAGT |
|  | R | GCCGATGATCTCTCTCAAGTGAT |
| IL-5 | F | CTCTGTTGACAAGCAATGAGACG |
|  | R | TCTTCAGTATGTCTAGCCCCTG |
| IL-13 | F | CAGCCTCCCCGATACCAAAAT |
|  | R | GCGAAACAGTTGCTTTGTGTAG |
| IL-10 | F | CTTACTGACTGGCATGAGGATCA |
|  | R | GCAGCTCTAGGAGCATGTGG |
| TGF-β1 | F | GAAGGCAGAGTTCAGGGT CTT |
|  | R | GGTTCCTGTCTT TGT GGT GAA |
| Foxp3 | F | ACCATTGGTTTACTCGCATGT |
|  | R | TCCACTCGCACAAAGCACTT |
| IL-6 | F | TAGTCCTTCCTACCCCAATTTCC |
|  | R | TTGGTCCTTAGCCACTCCTTC |
| IL-12p40 | F | TGGTTTGCCATCGTTTTGCTG |
|  | R | ACAGGTGAGGTTCACTGTTTCT |
